# What Do Dads Want in a Parenting Program? Improving Father Engagement in Parenting Education and Support

**DOI:** 10.3390/ejihpe15100202

**Published:** 2025-10-04

**Authors:** Amelia Rofe, Guy Hawkins, Emily E. Freeman

**Affiliations:** School of Psychological Sciences, The University of Newcastle, Callaghan, NSW 2308, Australia; guy.hawkins@newcastle.edu.au (G.H.); emily.freeman@newcastle.edu.au (E.E.F.)

**Keywords:** fathers, parenting programs, engagement, decision making

## Abstract

Father engagement in parenting programs is vital for positive child development and family wellbeing, yet fathers remain underrepresented in parenting programs. This study examines factors influencing fathers’ participation using two discrete choice experiments. Experiment 1 identified key attributes affecting engagement, including program content, cost, and delivery modality. Experiment 2 refined these insights, showing a preference for cost-free, face-to-face programs with practical activities. Findings highlight the importance of addressing financial barriers; offering flexible, relevant content; and designing father-friendly programs to improve accessibility and inclusivity. By prioritising fathers’ needs and preferences, parenting programs can better support child and family outcomes, fostering greater engagement and promoting equality across diverse family systems.

## 1. Introduction

*“If a community values its children, it must cherish their parents.”*

—John Bowlby

One of the most influential relationships a person will experience in their lifetime is arguably the relationship they share with their parents (Frosch et al., 2019). The parent–child relationship has a profound and pervasive impact on a child’s development, influencing a broad spectrum of areas including language and communication, executive function and self-regulation, sibling and peer relationships, academic attainment, and mental and physical health (Sanders & Turner, 2018). In this way, parenting is one of the primary tools to promote positive development across domains for children, including emotional, behavioural, and psychological wellbeing, and respond when these areas of functioning are compromised (Hoghughi, 1998).

In this context, parenting programs play a crucial role. ‘Parenting programs’ as a general term refers to programs that are designed to provide intervention and education to parents, equipping them with the skills and knowledge necessary to parent effectively, improve their relationship with their children, and promote positive developmental outcomes. The importance of these programs cannot be overstated; they are vital for ensuring that parents are adequately prepared to support their children’s growth and wellbeing as both preventative and intervention measures (Jeong et al., 2021). Throughout this article, universal parenting programs will be of focus, that is, programs that parents voluntarily engage with for preventative and early- and mid-stage intervention to address issues relating to parenting and the family system. Mandated programs, typically aimed at addressing acute issues in parenting or the family system, fall outside of the scope of the current article.

There is a distinct benefit to all members of the family system when parents engage in parenting programs. For fathers, participation in these programs has been shown to boost their confidence and sense of self-efficacy in parenting. Fathers who engage in these programs tend to develop more positive attitudes towards parenting, experience greater satisfaction in their parenting role, and report improved overall wellbeing alongside decreased stress (Bayse et al., 1991; Kaaresen et al., 2006; Wilczak & Markstrom, 1999). Their parenting partners benefit from improved relationship satisfaction and an increased sense of support from their male partners (Cookston et al., 2007), as well as prolonged gains from their own participation in parenting programs when their male partner is involved (Bagner & Eyberg, 2003).

The positive impact extends to children as well. The children of fathers who engage in parenting programs benefit from a more consistent approach to parenting from mum and dad, which leads to fewer behavioural problems, improved compliance, and stronger parent–child relationships (Webster-Stratton, 1985). Fathers who participate in these programs become more receptive to their children’s cues, communicate more effectively with them, and are more involved in their lives (Burgess, 2016; McBride, 1991; Levant & Doyle, 1983; Pugh, 2008). Furthermore, engagement in these programs reduces ineffective and harmful parenting practices, such as coercive parenting (McAllister et al., 2004), thereby enhancing both the short-term and long-term outcomes for children, including their social, psychological, and physiological health (Kendrick et al., 2013; Meek, 2007).

Despite the clear benefits of engagement in these programs, fathers remain a distinctly underserved population. On the Australian front, compared to mothers, fathers enrol in parenting programs at a rate five times lower, and their dropout rate can be alarmingly high, reaching up to 100% (Fletcher et al., 2011). This disparity exists on a global scale. In the United Kingdom, an evaluation of the Parenting Early Intervention Pathfinder, which was a government funded program promoting access to several evidence-based parenting programs, found that of 3575 parents engaged in a parenting program only 12% were fathers (Lindsay et al., 2008). Similarly concerning, an evaluation of a second government funded program promoting access to a universal parenting program for parents in the UK revealed that of 2956 participating parents, fathers only made up 9% (Lindsay et al., 2014). In the United States, an early review of parent engagement in parenting programs revealed that fathers make up just 13% of parenting program participants (Budd & O’Brien, 1982), putting in stark relief that this issue has not been appropriately ameliorated from the 20th century to now. More recently in the United States, Gonzalez et al. (2023) reveal in a review that many parenting intervention studies consistently reported father engagement below 50%, if they report it at all. This disparity, seen across decades and on an international scale, is concerning given the significant role effective parenting plays in ensuring the wellbeing of children and the positive outcomes that follow from engaged parenting. Addressing this gap in father engagement is therefore of critical importance.

However, current research falls short in this area. The broader trend in parenting program research has been to develop a wide range of programs targeting specific issues faced by children and their families (e.g., Bearss et al., 2013; Elder et al., 2011; Harrison et al., 2004; Nelson et al., 2016; Tonge et al., 2014; Wetterborg et al., 2019), with little focus on how to effectively engage parents, particularly fathers, in these programs. The research that does exist often asks fathers to consider isolated aspects of parenting programs when identifying factors that support or deter their engagement (e.g., Tully et al., 2017). Across this existing literature, several insights have been gained about factors that can support father engagement in parenting programs. These insights provide a solid foundation for the current article to further build upon. Freeman’s (2023) study clearly outlines a range of preferred program characteristics that work to align with fathers’ wants and needs when considering a parenting program. These include low cost or free of cost programs, program content that provide both activities and information to participants, face-to-face delivery of program content, and the opportunity to participate with other men. Whilst preliminary insights have been established in this area of research, undoubtedly further investigation is warranted given the pressing problem, and the need for the transition of research insights into practice in the family and child health and wellbeing industry. Not until research can be more effectively translated to practice recommendations can the issue of father under-engagement in parenting support services be further ameliorated.

To address this gap, the current study aims to explore the program features that facilitate or deter father engagement in parenting programs. This research takes a novel approach by employing a methodology that allows for deeper insights into fathers’ decision-making processes regarding their engagement in these programs. Two discrete choice experiments were conducted to determine fathers’ preferences for various parenting program features, considering these features within the context of holistic programs rather than as isolated factors. In Experiment 1, a global-level approach is taken wherein we gather broad, preliminary insights with the aim of informing the development of a more focused approach in Experiment 2. Through this process, we arrive at a set of actionable outcomes that can be clearly and easily translated into clinical practice.

A discrete choice experimental (DCE) design was used across both studies in this article (for overview, see Louviere et al., 2000). This methodology, traditionally applied in fields such as economics and cognitive and health psychology to examine individual’s decision making in regard to utilising health services, provides a structured way to examine decision-making processes in much greater detail. Its application to the context of child and family health, a field within which the current research is situated, is relatively novel, offering a unique lens through which to explore preferences in program engagement, and gain detailed insights that can be more effectively translated into practice recommendations.

For clarity, the term “experiment” is used throughout this article to describe the two studies. This aligns with standard DCE terminology, where participants are presented with hypothetical scenarios composed of systematically varied attributes. By observing how participants make trade-offs between these alternatives, researchers can infer preferences and estimate the relative importance of specific program features, from which valuable insights are gained.

## 2. Experiment 1

Experiment 1 aimed to explore the general features of parenting programs that influence men’s decisions to engage. This broad scope was reflected in both the study design and the participant sample, which included both current fathers and non-parent adult men. The intent was to gather wide-ranging insights from a population that is traditionally underrepresented in both parenting research and service provision.

The rationale for this inclusive recruitment approach within Experiment 1 was grounded in the urgent need to better understand and address male under-engagement in child and family health services. Fathers continue to be significantly underrepresented in parenting programs on an international scale, consistently making up the minority by a large margin in many programs. This issue is mirrored in the research literature, where fathers are often absent or marginalised in studies on parenting support, even when their perspectives are directly relevant (e.g., Gonzalez et al., 2023).

By including non-parent men alongside fathers in Experiment 1, we sought to amplify the voices of a broader population of men whose insights are rarely captured. While these participants may not yet be parenting, their willingness to engage in a study on this topic suggests that parenting is relevant to them—whether as a current consideration, future goal, or area of personal reflection. These perspectives are important, particularly given the lack of male representation in this field.

We acknowledge that this broad approach reduces the generalisability of Experiment 1’s findings, as insights from non-parents are not grounded in current parenting experience. However, we view this as a justified and deliberate trade-off, made in the service of promoting inclusivity and equity. Our aim was not to produce definitive conclusions about fathers as a clinical subgroup, but to create a strong foundation for further inquiry which we go on to do in Experiment 2, wherein we take a more targeted approach to the participant sample recruited.

It is important to highlight that Experiment 1 and Experiment 2 were designed as a series, with the first informing the second. Insights from the broader participant pool in Experiment 1 directly shaped the focus and attributes tested in Experiment 2.

As the research base focusing on identifying father’s preferences in relation to improving parenting program engagement continues to develop, we aim to take an exploratory approach to the investigation of attributes influencing the engagement decision in Experiment 1. Based on general trends across key literature, we do expect to see that fathers will show strong preferences for parenting program attributes related to cost and content (Frank et al., 2015; Freeman, 2023; Panter-Brick et al., 2014; Tully et al., 2017). Similarly, we expect facilitator qualities to be less consistently preferred, reflecting the distinct variation in fathers’ perspective towards this attribute across the existing literature (e.g., Frank et al., 2015; Freeman, 2023; Glynn & Dale, 2015).

## 3. Method

### 3.1. Design

The design of Experiment 1 adopted a broad, global-level approach to exploring the program characteristics, hereafter referred to as program attributes in line with DCE conventions, that influence men’s preferences for parenting programs.

A within-subjects experimental design using a best–worst scaling method was employed, whereby each participant completed the full set of best–worst choice tasks and provided preference data across all program attributes. Further details regarding the construction and implementation of this experiment are provided in the Materials section below.

### 3.2. Participants and Recruitment

Participants in Experiment 1 were eligible to take part if they were male, were aged 18 years or older, and resided within Australia. Recruitment occurred in 2024 via online advertising on social media platforms (i.e., Facebook). As compensation for participating, participants were invited to enter a prize draw to win one of three $60 digital gift cards.

Notably, prior participation in a parenting program was not required for eligibility. This was a deliberate design decision. Restricting the sample to men who had already accessed parenting programs would likely have biased the sample towards those who already feel able to access support and navigate available services. In contrast, including men who had not previously engaged allowed the study to capture perspectives from those who may feel excluded, unsupported, or unaware of relevant services, representing groups whose insights are essential for designing more accessible and inclusive parenting support.

The authors consider the inclusion of both engaged and non-engaged participants a strength of the study, allowing for a more comprehensive understanding of the barriers and enablers to program engagement. This inclusive approach supports the broader aim of informing service design in a way that better meets the diverse needs of fathers, mothers, and other caregivers.

A total of 54 participants began the experiment, with 45 completing all tasks in full. Concerns about sample size and statistical power must be considered within the context of DCE methodology, which differs from traditional survey-based designs. DCEs analyse how participants make trade-offs between different program attributes by presenting them with systematically varied choice tasks. This method allows researchers to estimate the relative importance of each attribute and model decision-making preferences in detail.

Notably, DCEs can yield valid results with smaller sample sizes than other quantitative methods, since DCEs obtain repeated observations for each participant for each measured construct. Many DCEs successfully operate with sample sizes between 100-200 or lower, particularly those with focused populations of interest. For instance, a widely cited review by de Bekker-Grob et al. (2015) reported that 32% of health-related DCEs used fewer than 100 participants, particularly where the number of choice tasks and attributes was modest.

Further, our sample size targets were informed by expert guidance in the field. Lancsar and Louviere (2008) note that reliable models can often be estimated with as few as 20 respondents per version of a questionnaire, particularly where the goal is preference estimation rather than subgroup comparisons, congruent with de Bekker-Grob and colleagues’ recommendations. Johnson and Orme (2003) and Orme (1998) also suggest that sample size adequacy depends on the number of choice tasks, alternatives, and model parameters rather than absolute numbers alone. These considerations were integral to our planning, especially given the well-documented issue of father under-engagement in both service provision and research.

Within this context, we considered 45 complete responses in Experiment 1 to be acceptable, albeit at the lower bound of typical DCE studies. We acknowledge this as a limitation, particularly with respect to generalisability, and this is further discussed in the General Discussion. However, we maintain that these findings provide valuable preliminary insights and served their intended purpose of informing the more focused Experiment 2, which benefited from a larger and more targeted sample, discussed further on in this article.

### 3.3. Materials

All materials used in the research presented across the current article are described in full within the article; no protected or proprietary materials were used.

#### 3.3.1. Demographic Questionnaire

Participants completed demographic questions asking about their age, education, employment, ethnic background, relationship status, number of children, and previous experience participating in parenting programs. These demographic characteristics were divided into levels, outlined in Table 1. Age included four levels, education and employment included six levels each, ethnicity included eight levels, relationship status included three levels, number of children below 18yrs of age included four levels, and previous engagement in a parenting program was a dichotomous yes/no question.

#### 3.3.2. Experiment

**Figure 1:**
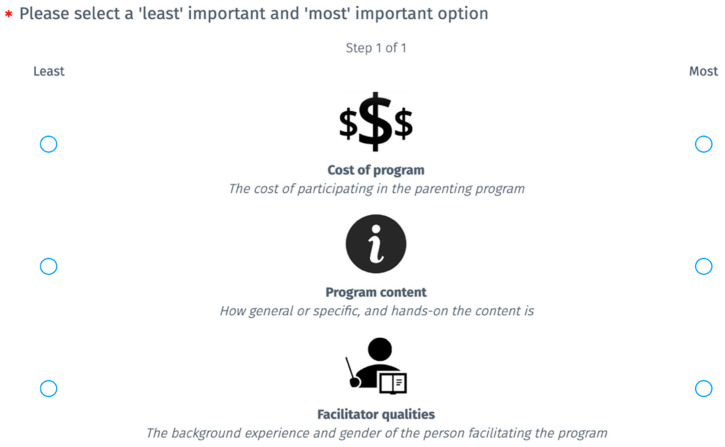
Example of a question from Experiment 1. Note. The term ‘best’ maps to most preferred option, and the term ‘worst’ maps to least preferred option out of the options presented to the participant. Red * is part of the website formatting, and simply denotes that the participant is required to answer each question when they enrol in the study. The website - prolific - has this function to ensure participants don’t accidentally skip a question when scrolling through, It is not meaningful for the purpose of this article.

In Experiment 1, participants’ preference towards six global-level parenting program attributes in the context of considering engagement in a parenting program were explored. These six attributes are outlined in Table 2 (Experiment 1). To capture these preferences, we used the Best–Worst Scaling method (Louviere et al., 2015). This method presents participants with a series of options, and for each option they are asked to indicate both the ‘best’ and ‘worst’ attribute. When a participant identifies an attribute as the ‘best’, they are indicating that this is their most preferred attribute, whilst an attribute identified as the ‘worst’ is their least preferred out of the attributes presented to them in that hypothetical option. A D-optimal design was used for the Best–Worst Scaling questions, which optimises the amount of information gained while reducing the number of choices participants are required to make. Each question presented participants with three of the possible six attributes (see Table 2). Each of the six program attributes were presented to participants ten times each across the 20 questions, and each program attribute was presented with every other program attribute an equal number of times. The order of the 20 questions was randomised across participants to prevent fatigue effects. An example question is displayed in Figure 1.

### 3.4. Procedure

Participants completed the experiment on the QuestionPro survey hosting website. The experiment was anonymous, and participants provided consent to participate at the beginning of the experiment. If they did not indicate their consent, they were automatically exited from the QuestionPro site. Participants first completed the demographic questions followed by the DCE.

### 3.5. Data Analysis

Descriptive statistics were calculated for the demographic data and are reported in Table 1.

A Best–Worst scaling standardised score was calculated for each participant for each of the six program attributes as a measure of preference (least preferred attribute to most preferred attribute). This standardised score is calculated by subtracting the number of ‘worst’ votes (i.e., participants voted this attribute as least preferred X number of times) for a single attribute from the total number of ‘best’ votes (i.e., participants voted this attribute as most preferred X number of time) for that same attribute. This value is then divided by the total number of appearances of that attribute to participants (Louviere et al., 2015). This formula (best-worst)/appearances results in a value that ranges from −1 to 1 for each attribute and reflects the overall strength of participants preference for that attribute. Scores towards 1 indicate that this attribute was more important in participants’ decision making, while scores towards −1 indicate this attribute was not important/had little influence on participants’ decision making when considering engaging in a parenting program (Flynn et al., 2007). Practically, this data allows us to consider how much different program attributes influence individuals’ decision to engage in a program, (e.g., if the program has an associated enrolment cost, for instance, how much is this a make-or-break factor for men considering enrolling in that program or not).

To analyse differences in preferences between the six program attributes, a one-way repeated measures ANOVA and subsequent post hoc tests were performed.

## 4. Results and Discussion

Participants who did not complete the survey after beginning were excluded, leaving a sample of forty-five data points, which comprise the following analysis.

### 4.1. Demographic Characteristics

Descriptive statistics derived from the demographic data are presented in Table 1. Participants ranged in age from 18 to 52 years (Mean age = 28 years, SD = 11 years). The majority of participants were between 18–30 years of age (71%), with most having achieved a high school diploma (38%) and enrolling in university (29%). Similarly, most participants were employed part-time (29%) or full-time (38%). Seventy-one percent of participants were from a Caucasian/Australian background, and 60% reported being single/never married. Just under half (47%) of participants reported having 0 children under the age of 18 years, the remaining 53% (cumulatively) reported having one or more child currently under the age of 18 years.

Most participants (93%) reported having never engaged in a parenting program before, with three participants reporting previous engagement. Across these three participants, previous parenting programs included 1, 2, 3 Magic, which is a program designed to help parents and caregivers manage challenging child behaviours, particularly those of children aged 2–12, and focuses on teaching parents how to respond to unwanted behaviours with a simple “1-2-3” counting method and how to coach children through their emotions. The second program participants reported previous involvement with was a free parenting education and support service delivered by the New South Wales public health system in Australia, likely referring to one of the ‘Positive Birth and Parenting workshops’ offered by the public health system (although this was unnamed by the participant in the current experiment). One participant reported having previously engaged in a parenting program, but did not describe or indicate which program he had engaged in. This trend is in line with the rate reported across the literature, with men reportedly engaging significantly less than mothers (Fletcher et al., 2011).

### 4.2. Parenting Program Preferences

The average standardised score for each of the six attributes was calculated using the method described in Section 3.5 and is reported in Table 3. General trends for participants’ preferences across the six attributes can be observed, with program content being considered the most important attribute by participants in the decision to engage in a parenting program, whereas program length surprisingly had very little influence on participants’ decisions. This was followed by facilitator qualities similarly being of little importance to participants decision making. Style of participation and program cost were considered moderately important to participants, and delivery modality was of neutral importance to participants.

Table 3 can be interpreted by understanding that rated strength of preference of each attribute moves from least preferred (meaning the attribute does not play a part in decision making much at all) on the right, moving towards the middle which represents participants being mixed about whether these attributes were important for their decision making process, to the far left where these attributes play a very important part in participants’ decision making and are considered the most preferred (as in, the participant puts the most weight on these attributes when considering engagement in a parenting program).

A one-way repeated measures ANOVA was conducted to determine whether there was a statistically significant difference in participants’ preferences across the six program attributes. Mauchley’s test indicated that the assumption of sphericity was violated (χ^2^ (14) = 32.47, *p* = 0.003); therefore, degrees of freedom were corrected using the Greenhouse–Geisser estimate of sphericity (ε = 0.76). The results of the corrected ANOVA revealed that there was a significant effect of program attribute on participant’s preference between at least two of the attributes (*F*(3.773,166.026) = 10.34, *p* < 0.001).

Post hoc tests (using the Holm correction to adjust p) indicated that participants consistently rated program length as less important than any of the five other attributes (all pairwise comparisons *p* < 0.05). Further, participants rated that program content was significantly more important than both facilitator qualities (*p* = 0.002), and delivery modality (*p* = 0.04).

A consideration of the general preference trends, results of the one-way repeated measures ANOVA, and post hoc testing revealed participants placed the highest importance on the content of the parenting program in their decision to engage, followed by the cost, and then the style of participation. Results indicate that these three attributes do impact men’s decisions regarding engaging in a program, and they look at these attributes in a potential program to ensure what is on offer (in regard to these attributes) meets their needs.

The delivery modality was of neutral importance, likely indicating that the sample was split between those who found this attribute important to consider, and those who did not care about delivery modality.

Finally, participants found the length of the program unimportant when considering engaging in a hypothetical program, denoting the sample largely agreed they did not care about this attribute in their decision-making process. Similarly, facilitator qualities were of little importance to participants decision making. Practically, this likely suggests that when men come across a program that effectively meets their needs regarding their most preferred attributes (i.e., program content offered, cost of the program, and the style of delivery), then they are less concerned about how long the program spans for or who is facilitating it.

## 5. Experiment 2

Much of the current research on effectively engaging fathers in the field of family and child health, particularly in recruiting and retaining more fathers in parenting programs, tends to examine fathers’ preferences in isolation. Experiment 1 continued this trend, contributing insights from an Australian male sample. This approach, while contributing to the growing field of father-focused research, does not effectively translate into practice, as an individual’s decision to engage in a parenting program is complex and multidimensional, and needs to be considered as such.

Experiment 2 built directly upon the findings of Experiment 1 by incorporating the program attributes that participants had indicated a preference for, thereby introducing an important element of co-design from the population of interest. In this second experiment, a more specific and focused approach was adopted to gain deeper insight. While Experiment 1 deliberately employed a broad sampling strategy which invited both current fathers and non-parent men to reflect on factors influencing their engagement decisions, Experiment 2 was limited to current fathers of children under the age of 18 years. This shift in focus was a purposeful decision made by the researchers.

A central aim of the current article is to contribute to the growing body of work seeking to improve equality and accessibility within parenting support services for fathers, who remain significantly under-engaged and under-served in this space. Casting a wide net in Experiment 1 allowed the inclusion of diverse male perspectives, especially from individuals who may be considering fatherhood as well as fathers who have not yet accessed services. In this way, the intention was for Experiment 1 to provide a broad theoretical foundation that could inform the development and design of Experiment 2 whilst providing a voice to fathers and men. Experiment 2 represents the focal point of the current article, offering insights that are more directly applicable to fathers specifically.

A second way in which we increased the detail and specificity in Experiment 2 was in the methodological approach. In this discrete choice experiment, attribute levels were introduced, and fathers were asked to consider multiple levels of each attribute together in the context of a hypothetical, holistic parenting program, compare the hypothetical programs, and trade-off the components of each program to select their preferred overall program. This approach allows for deeper insight into how fathers make decisions about participating in parenting programs and how different aspects of a program could be integrated for the father considering his engagement in a program.

In the interest of incorporating elements of co-design, and navigating the risk of cognitive fatigue, which is heightened in Experiment 2 as further choice options are added through the inclusions of attribute levels, resulting in more complex choice tasks for fathers to complete, insights about program attributes that were revealed to have little impact on fathers decision making regarding program engagement were used to inform the design of Experiment 2 (Krosnick, 1999).

Program content, cost, and style of participation were identified as key factors fathers consider in their decision to engage, and the sample was largely split regarding delivery modality, indicating this factor may indeed be important to some but less important to others. Conversely, facilitator qualities and program length were revealed as attributes that did not factor into the decision to engage in a parenting program.

These findings align with broader trends in the existing literature on father engagement in parenting programs. Studies have consistently reported mixed results regarding the influence of facilitator characteristics (e.g., Frank et al., 2015; Freeman, 2023; Glynn & Dale, 2015), suggesting that fathers do not express a strong or consistent preference for facilitator background, style, or characteristics. While it is possible that facilitator preferences are highly individualised, the results of Experiment 1 suggest that, for most men, facilitator qualities are simply not a salient factor when considering whether to participate. In light of this, and with the goal of reducing participant burden, facilitator qualities were excluded from Experiment 2, as its inclusion was unlikely to generate meaningful new insights beyond those already established in the literature and confirmed in Experiment 1.

The lack of importance of program length was a more unexpected outcome. In the broader parenting program literature, interventions commonly span 8–12 weeks (e.g., Bagner, 2013; Berge et al., 2010; Feinberg et al., 2010; Hoofdakker et al., 2014; Roberts et al., 2006; Sanders et al., 2000; Wilson et al., 2012). However, this standardised duration may not reflect fathers’ actual preferences. In contrast to the structure of many existing programs, emerging research that focuses specifically on fathers’ preferences has found that shorter programs, typically 2–4 weeks in duration, are preferred (Freeman, 2023). Program length was ranked as the least important attribute in Experiment 1. Given this, and to further reduce participant fatigue in Experiment 2, program length was also excluded from the final design. The remaining program attributes and attribute levels are outlined in Table 2.

By focusing only on the attributes identified as most relevant to men and fathers, Experiment 2 aimed to deepen insight into the decision-making processes regarding parenting program engagement. We hypothesised that practical activities would emerge as the most preferred content style, followed by specific and then general information, while cost preferences will show an increasing trend favouring free or low-cost programs over more expensive options. Additionally, we expected that fathers would prefer group settings with other fathers, followed by groups of male and female parents, and then participating individually in programs. We finally hypothesised that fathers would most prefer participating in programs face-to-face, followed by Telehealth (synchronous), and prefer online participation (asynchronous) least.

## 6. Method

### 6.1. Design

A within-subjects discrete choice experimental design was employed in which all attributes and attribute levels were presented to each participant across their completion of the online experiment.

### 6.2. Participants and Recruitment

Participants were eligible to take part if they were male and aged 18 years or older, were a current parent of a child under the age of 18 years, and resided within Australia. Participants in Experiment 2 were recruited during 2024 through advertising on social media (Facebook), internally through the authors’ institution, and via the online experiment platform Prolific. A total of 102 participants were recruited across these three platforms. Fifty-two participants were recruited on the Prolific platform, 29 participants were recruited from social media advertising, and 21 were recruited internally through the author’s institution. In line with Experiment 1, participants were not required to have previously engaged in a parenting program.

### 6.3. Materials

#### 6.3.1. Demographic Questionnaire

Participants in Experiment 2 completed the same set of demographic questions used in Experiment 1 to allow a comparison of samples.

#### 6.3.2. Experiment

**Figure 2:**
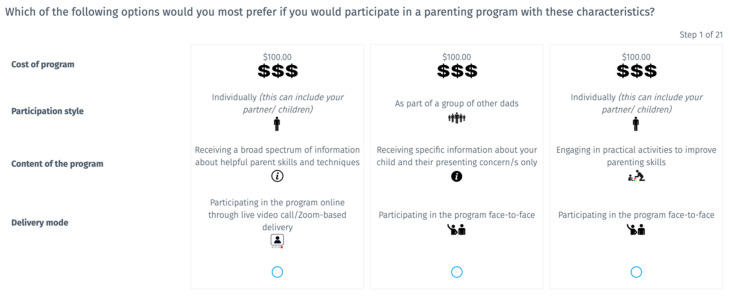
Example of a question from Experiment 2.

Four of the original six program attributes comprised Experiment 2; these included cost, delivery modality, style of participation, and program content. Three levels were developed for each of these attributes informed by features of current validated parenting programs. Each attribute and their levels are outlined in Table 2. Each participant completed 21 choice sets, which included three hypothetical parenting programs comprising one level of each of the four attributes. Participants were required to select their most preferred hypothetical parenting program from the set of three options. An example question is displayed in Figure 2 to illustrate this design. The trials had a randomised design, meaning that each hypothetical parenting program choice comprised a random selection of levels from each of the four attributes. This randomised design was used to reduce the risk of systematic bias in the presentation of attributes to participants and to reduce overall cognitive load by allowing the use of a briefer set of decision tasks.

### 6.4. Data Analysis

Descriptive statistics were calculated for the demographic data, which is reported in Table 1.

A multinomial logistic regression was conducted to analyse the data from the decision tasks. The goal was to understand the impact of four key predictors on participant’s decision making when considering engaging in parenting programs: program cost, participation style, program content, and delivery modality. To this end, the regression was set up to predict the hypothetical parenting program chosen (i.e., option 1, 2, or 3) for each of the 21 trials completed by each participant. We dummy coded the three levels of each attribute for inclusion in the regression model, with one level of each attribute set as the reference level. We refer to the dummy coded regression coefficients as the utility of the attribute level, which can be thought of as a measure of how much participants preferred that attribute level, with larger and more positive values representing greater preferences. We then calculated the utility of an option as the sum of the component utilities of the attribute levels that compose the option. We repeated this for each option in each trial, and then across all trials and participants. This is an additive utilities representation that is standard practice in the analysis of discrete choice experimental data. The Apollo package in the R programming language was used for model estimation (Hess & Palma, 2019). Given the complexity of human decision-making, a random effects model was employed to account for the variability in how different participants weighed the attributes when making choices.

**Figure 3:**
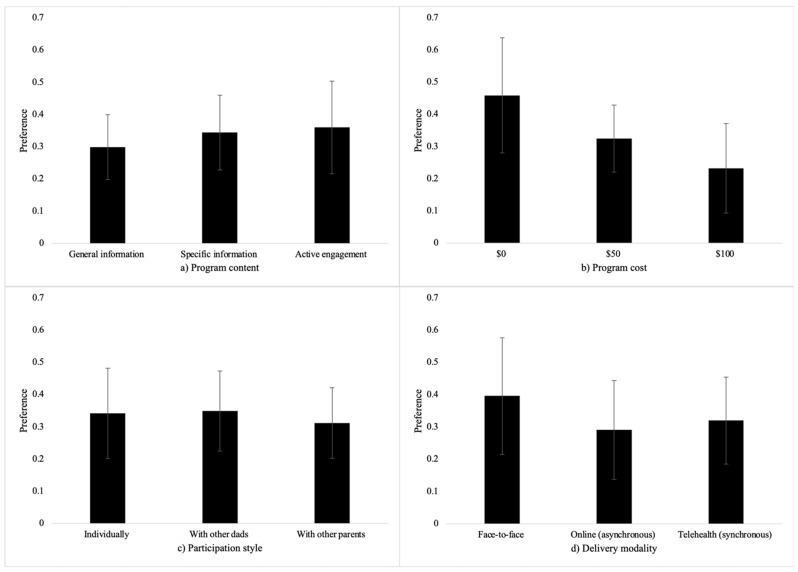
(**a**). Fathers’ preferences across attribute levels of program content. (**b**). Fathers’ preferences across attribute levels of program cost. (**c**). Fathers’ preferences across attribute levels of participation style. (**d**). Fathers’ preferences across attribute levels of delivery modality.

To help guide readers, our results are stepped across three key stages. Firstly, we present the preliminary trends in father preference that appear across the four parenting program attributes, Figure 3 helpfully visualises these trends.

Secondly, we checked these trends utilising the above-described multinomial logical regression, allowing us to confirm the presence of these trends beyond visual inspection and examining significance of these trends.

Thirdly, we attempt to translate our findings to applicable practice recommendations for the field of child and family health and wellbeing. To do this, we adopt a model-based inference approach (described in detail below) to identify the preferred holistic parenting program design that works to meet fathers’ needs and preferences as wholly as possible. In this section, two potential parenting programs are presented for consideration.

### 6.5. Results and Discussion

Participants who did not complete the survey after beginning were excluded, leaving a sample of 97 participants, which comprise the following analysis.

### 6.6. Demographic Characteristics

Descriptive statistics derived from the demographic data are presented in Table 1. Participants in Experiment 2 ranged in age from 18 to 74 years (Mean age = 39 years, SD = 10 years), making the sample in Experiment 2 comparable to that in Experiment 1, with some increased representation for older aged fathers. Demographic categories established in Experiment 1 were used in Experiment 2. All levels of education, employment and relationship status were represented. Many participants were university educated, with a large number having achieved a postgraduate level of study, resulting in participants in Experiment 2 being, on average, a more educated sample than Experiment 1. Some research in the field has shown that parent’s level of education influences parenting, and is related to socioeconomic status, which similarly has been found to have a significant impact on parents’ access to parenting supports. Further to this, the majority of the sample reported full time employment, which similarly may represent a more affluent or resourced sample overall (Chau et al., 2023; Crawford et al., 2016). Also of note, the majority of participants in Experiment 1 reported being single, while the majority of participants in Experiment 2 reported being married or in a de facto relationship.

In sum, the sample was from a Caucasian/Australian background, educated, in a relationship, and often had two or more children. This sample reported having previously engaged in a parenting program at a much higher rate compared to Experiment 1, with 25 out of the total 97 participants (26%) reporting previous engagement, however it may be pertinent to consider to increased and more focused sample size in Experiment 2 here.

### 6.7. Parenting Program Preferences

Summary statistics were calculated to explore preliminary trends in fathers’ preferences for attributes of parenting programs, focusing on program cost, participation style, program content, and delivery modality. Marginal choice proportions for each attribute are displayed in Figure 3. The marginal choice proportion shows how often a specific attribute level (e.g., cost $0) was in the parenting program that was chosen as the preferred option divided by how often a program with that level included was presented. For example, if in 16 choice sets at least one program was presented that had a $0 cost, and in 10 of those choice sets, the participant chose the program with the $0 cost as their preferred option, then their marginal choice proportion for a $0 cost parenting program is 10/16 = 0.625.

A clear pattern of preference was observed across the levels of program cost, with participants demonstrating a clear preference for free programs, followed by a preference for programs costing $50, and the lowest preference for programs priced at $100. In contrast, participation style showed little variation, with each of the three levels (individual participation, participation with other dads, and participation with other parents) clustered around chance selection, suggesting no strong preference for any specific style. Small amounts of variation were observed for both program content and delivery modality, indicating that fathers exhibited some preferences for these attributes. Figure 3 visualises these preference patterns, with larger values indicating stronger preference for certain attribute levels and values closer to 0 indicating weaker preferences.

### 6.8. Multinomial Logit Analysis

#### 6.8.1. Model Fit

A multinomial logistic regression was conducted to examine fathers’ preferences for parenting program attributes, including program cost, participation style, program content, and delivery modality as predictors. Each attribute had three levels (see Table 2). The model fit was assessed using the adjusted Rho-squared, R^2^ = 0.1802, meaning the model explained approximately 18% of the variation in fathers’ preferences for parenting programs based on the included predictors (program cost, participation style, program content, and delivery modality) and the random effects structure. For context, R^2^ values in DCEs are not directly comparable to those in linear regression, as they are based on differences in log-likelihoods rather than explained variance in the dependent variable. In fact, R^2^ values between 0.1 and 0.3 are generally accepted as representing good model performance in DCE literature (Louviere et al., 2000). Values closer to 0.2, as seen in the current model, suggest a meaningful and interpretable level of explanatory power, particularly given the inherent complexity and multidimensionality of human decision-making. Notably, when a random effects structure was not applied to the model, the fit was substantially decreased (R^2^ = 0.08), suggesting considerable individual differences in preferences, with explanatory power increased when these are incorporated into the model. Parameter estimates for the model using the random effects structure are reported in Table 4.

#### 6.8.2. Pairwise Comparisons

Following assessment of the model fit, we next compared the levels of each attribute with one another using the Delta method (Daly et al., 2012). The results indicated significant differences between cost levels. Specifically, participants were more likely to prefer free programs over those costing $50 (β = 0.74, *t* = 6.24, S.E. = 0.12, *p* < 0.001) and $100 (β = 1.70, *t* = 5.89, S.E. = 0.29, *p* < 0.001). Additionally, programs priced at $50 were preferred over those costing $100 (β = 0.96, *t* = 3.76, S.E. = 0.25, *p* < 0.001).

Regarding participation style, no significant differences were observed between individual participation and participation with other dads (β = 0.13, *t* = 0.71, S.E. = 0.18, *p* = 0.478), individual participation and participation with other parents (β = 0.16, *t* = 1.06, S.E. = 0.15, *p* = 0.289), or between participation with other dads and other parents (β = 0.03, *t* = 0.22, S.E. = 0.15, *p* = 0.826).

For program content, fathers showed a significant preference for programs providing specific information over those with general information (β = 0.36, *t* = 2.81, S.E. = 0.13, *p* = 0.005), and similarly a preference for programs providing activities and practical skills over those providing general information (β = 0.50, *t* = 2.59, S.E. = 0.19, *p* = 0.01). However, there was no significant difference between programs providing activities and practical skills and those providing specific information (β = 0.14, *t* = 0.63, S.E. = 0.23, *p* = 0.529).

Finally, fathers preferred face-to-face delivery over both online (β = 0.70, *t* = 2.74, S.E. = 0.26, *p* = 0.006) and telehealth delivery (β = 0.79, *t* = 2.94, S.E. = 0.27, *p* = 0.003). No significant differences were found between online and telehealth delivery modalities (β = 0.08, *t* = 2.27, S.E. = 0.32, *p* = 0.787).

### 6.9. Preferred Parenting Program

Building on insights from the multinomial logit analysis, a model-based inference approach was next employed to identify the holistic parenting program with the highest predicted preference among fathers. This method aimed to provide further insights into how parenting programs can use a more ‘father-friendly’ design to improve father engagement. By refining the model-predicted outcomes from ‘option 1’ in ‘option 2,’ the goal was to bridge the gap between theory and practice and provide research-based guidance for how the design and delivery of parenting programs can effectively work to service and engage fathers.

Using model predictions, we calculated the predicted probabilities of selection for each of the 81 unique parenting programs possible, generated by combining four at-tributes, each with three levels. This yielded probabilities (p) and cumulative probabilities (*p*_cumulative_) for each of the 81 options of hypothetical parenting programs that could be presented to participants, which functionally revealed what percentage of the predicated choice share of the 81 possible programs each option accounted for.

#### 6.9.1. Program Option 1

The top three preferred parenting programs all featured $0 cost, practical activities as content, and face-to-face delivery, differing only in participation style (i.e., individual participation, participation with other dads, and participation with other parents). This finding aligns with prior analysis, which showed no significant differences in fathers’ preferences for delivery modality. These top three programs accounted for approximately 10% (*p*_cumulative_ = 0.099) of the total predicted choice shared among the 81 options. Table 5 presents the top 10 preferred programs, based on model-predicted choice shares.

#### 6.9.2. Program Option 2

As fathers’ preferences were heterogeneous for participation style, indicating that fathers do not hold a strong preference towards this attribute as compared to other attributes considered in the current experiment, further exploratory analysis was conducted by excluding this attribute from consideration. This decision is built on the assumption that, as fathers’ preference for participation style significantly varied, this attribute could vary in practice whilst still prioritising fathers’ preference in parenting program design and administration.

A revised predicted choice share was calculated with participation style excluded (specifically, we marginalised over the participation style attribute). In this revised analysis, the parenting program identified above ($0 cost, practical activities as content, and face-to-face delivery) again accounted for approximately 10% of the predicted choice share. The second most preferred program similarly included cost $0 and face-to-face delivery but differed by including specific information as the preferred program content. Considering these top two programs together, approximately 18% (*p*_cumulative_ = 0.181) of the predicted choice share is captured. The top 10 preferred options from this second round of exploratory analysis of predicted choice shares are reported in Table 6.

Based on the analysis, Program Option 2, a parenting program offered at no cost, delivered face-to-face, and incorporating active engagement with specific information, emerged as the most preferred option, capturing up to 18% of the predicted choice share. Drawing on principals outlined when discussing model fit above, this is an encouraging result and represents a meaningful portion of variability in fathers’ decision making. This outcome highlights that cost and delivery modality are the most influential attributes in fathers’ decision-making. Free programs were overwhelmingly favoured, and face-to-face delivery was more popular compared to online (asynchronous) or telehealth (synchronous) delivery options. Participants showed a strong preference for programs integrating active engagement and specific information. Participation style (individual, with other dads, or with other parents) appeared less important, suggesting that fathers are flexible about who they participate in parenting programs with. This aligns with pairwise comparisons, further supporting Program Option 2 as the best choice for fathers when deciding to engage in a parenting program.

## 7. General Discussion

Through two discrete choice experiments, the current study explored how men and fathers consider parenting program attributes when deciding whether to participate. The aim was to better understand which program features support better engagement from this population, who remain significantly under-serviced in the field of child and family health and wellbeing. In this way, we hoped to contribute towards the development and design of more father-friendly parenting programs in the future. In doing so, the study contributes to the developing body of literature focused on improving fathers’ experiences with, and access to, child and family health and wellbeing services.

Experiment 1 provided a broad overview of men and fathers preferences relating to general program attributes. Program content emerged as the most important factor that participants considered in their decision to engage in a program. This was followed by program cost and participation style. Delivery modality appeared to hold neutral importance, suggesting participants were divided in how much this attribute played a part in the decision to engage, while facilitator qualities and program length were revealed as playing very little part in shaping engagement decisions. Building upon these findings, Experiment 2 introduced more detailed choice tasks and focused on a more specific participant sample, of current fathers of children under the age of 18 years, to gain a deeper understanding of fathers’ decision-making. Findings revealed that fathers consistently preferred free programs that offered practical activities and were delivered face-to-face. These findings underscore the importance of designing accessible and relevant programs that meet fathers where they are.

This article provides important insights about how to practically design parenting support services with fathers’ needs and preferences in mind. The value of these findings lies not only in improving fathers’ engagement in parenting programs and in doing so supporting men as parents, but in strengthening the entire family system. Fathers contribute uniquely and meaningfully to children’s development, and when they are supported to parent competently and confidently, child outcomes and the wellbeing of all family members improve. Fathers’ often more physical, playful, and risk-oriented interactions help broaden children’s social, emotional, and cognitive capacities (Braungart-Rieker & Planalp, 2016; Dumont & Paquette, 2012; Freeman & Robinson, 2022; Robinson et al., 2021). Moreover, research has shown that fathers meaningfully contribute to children’s language development (Leech et al., 2013; Pancsofar et al., 2010). When fathers access support and their parenting skills improve, their capacity to provide support to their partners in turn improves, enhancing relationship satisfaction and wellbeing for mothers (deMontigny et al., 2020; Kwok et al., 2013). Failing to engage fathers in available programs means that they often miss out on the same skill-building and support that mothers are more likely to access, ultimately limiting the benefits children and families could receive through better-supported paternal involvement.

While these findings offer meaningful implications, several limitations must be acknowledged. First, methodological choices in Experiment 1, including the broad sampling of both fathers and non-fathers, limited the generalisability of the findings. This decision was made deliberately to align with the study’s overarching aim of improving accessibility and inclusion for men in general within the space of child and family health and wellbeing, a population historically underrepresented in both parenting support and the research conducted in this field. However, readers should interpret the findings of Experiment 1 with caution, as they do not represent the lived experiences of only active fathers.

Second, although Experiment 2 included only current fathers and provided more targeted insights, the sample was relatively homogenous. The majority of participants were Caucasian, educated, and gainfully employed Australians. These demographic characteristics are known to impact both parenting capacity and access to support resources (Kalil & Ryan, 2020; Roubinov & Boyce, 2017). Through the lens of Maslow’s (1943) hierarchy of needs theory, it becomes clear that fathers from more disadvantaged backgrounds who may experience economic, psychological, or practical stressors are likely to not only be deterred by such practical and societal barriers, by lack the psychological and emotional resources to access parenting programs, despite the desire to improve parenting skills and be actively involved in their fathering role from a competence and confident stand-point.

Another noteworthy limitation involves the prior engagement of participants in parenting programs. Fathers in Experiment 2 reported much higher rates of previous program participation compared to those in Experiment 1. While this is partially explained by the inclusion of non-parent men in Experiment 1, it also raises a broader concern: perhaps the fathers who are most under-served by parenting services remain entirely unrepresented in the research. The fathers who volunteer to participate in research like this one may be those who already demonstrate interest and motivation to engage, thereby continuing to under-engage the voices of those who feel most alienated or marginalised by the child and family health and wellbeing system. This concern is vital for future research to consider when striving to develop solutions for under-engagement. A strength of the current research arises here, in that we were able to engage men and fathers who report not having previous engage in parenting programs, likely collecting valuable insight from these participants who previous have access parenting support.

Despite these limitations, the findings offer key implications for both research and practice. The identification of cost, content, and modality as critical engagement factors supports the development of more accessible and inclusive parenting services. Working to reduce or eliminate program fees may lower significant barriers to entry. Offering face-to-face options, alongside flexible alternatives (e.g., online or hybrid formats), can cater to fathers with varying preferences and schedules, practical accessibility considerations such as these align closely with key findings in the field of literature (Pfitzner et al., 2017) Prioritising practical, relevant content over generic or didactic material may further enhance engagement and program effectiveness. Despite many of these recommendations seeming like small adjustments, they are able to improve inclusivity and accessibility for fathers on a broad scale. Whilst fathers are not actively discouraged from engaging in available parenting support, social, practical and structural factors work to decrease how accessible and welcoming parenting support services appear for fathers. For instance, the marketing material for many parenting support services and programs refer to ‘parents’ but display images of mothers with children, delivering the message that the program is directed towards the mother as the primary caregiver (e.g., Glenelg Shire Council, n.d.). Further, social factors such as gendered biases and systemic traumatisation can silently shape father-specific engagement in parenting programs. As noted earlier in this article, our research focused on universal parenting programs in which parents voluntarily participate. However, there is also a mandated pathway into parenting services, wherein parents are recommended or required to complete parenting remediation through involvement in the family court system, often in contexts of acute family dysfunction or child safety concerns.

In considering the statistics, it is arguable that gendered differences, and, arguably gendered biases, significantly affect fathers’ experiences within the family court system and thus with involuntary parenting services. For example, in Australia, mothers are granted sole care of the children, or between 87–99% of the available parenting time, in 39% of cases. In contrast, fathers are granted sole parental responsibility or 87–99% of parenting time in only 3% of cases (Australian Institute of Family Studies, 2019).

While it is essential to interpret these figures alongside the critical issue of safety in the context of domestic and family violence, they nevertheless highlight a broader social reality, that awareness of such disparities can shape how men collectively perceive and engage with parenting services. For some, these services may be seen less as supportive and more as punitive, reinforcing a perception that the important role of fathers in child development is undervalued or overlooked. This perception, in turn, may influence the willingness of fathers to engage with parenting programs in a meaningful way, or see it as hopeless or pointless from the outset because of the ingrained message that fathers are not as valuable as mothers in parenting.

Factors such as this undoubtedly contribute to the low rate of father engagement in parenting programs, and then when fathers do engage and find themselves in a group primarily comprising mothers (i.e., Lindsay et al., 2014; Tully et al., 2017), and naturally receive parenting support delivered with a majority audience of mothers in mind by program facilitators, we then are able to understand the high dropout rates of fathers from the programs they do engage in (Fletcher et al., 2011).

This research adds to a growing consensus that failing to design inclusive, equitable parenting support can undermine the very purpose of these interventions. Resources that do not account for fathers’ needs and lived experiences may ultimately exacerbate disparities within family systems. The same applies to other under-serviced populations. For instance, Van Esch and De Haan (2017) and Panter-Brick et al. (2014) illustrate, and Haslam and Mejia (2018) further discuss how culturally diverse families often feel excluded from mainstream parenting programs due to poor cultural tailoring and insensitivity in program delivery. An inclusive and equitable approach to program design that recognises and responds to different experiences of marginalisation and under-servicing is crucial for improving outcomes across the full spectrum of modern family systems.

This study supports the urgent need to reconsider how we approach parenting program design, delivery, and implementation to be inclusive, flexible, and attuned to the diverse needs of all parents. While this research focuses on fathers, it illustrates broader systemic shortcomings in how parenting support is conceptualised and delivered. Future research must continue to explore the unique needs of all caregivers including mothers, fathers, co-parents, and extended kin from diverse backgrounds and with diverse experiences if parenting programs are to reach their full potential and thus provide the benefit to family systems they are designed to. By addressing barriers and fostering inclusion, we can better serve the evolving needs of today’s families and ensure that all children benefit from engaged, informed, and supported caregivers.

## Figures and Tables

**Table 1:** Participant demographic data from Studies 1 and 2.

Demographic Domain	Demographic Category	Experiment 1 (N = 45) *N (%)*	Experiment 2 (N = 97) *N (%)*
Age	18–30	32 (71)	18 (19)
	31–40	4 (9)	38 (39)
	41–50	6 (13)	29 (30)
	51+	3 (7)	12 (12)
Education	Less than a high school diploma	3 (7)	5 (5)
	High school graduate, diploma or the equivalent	17 (38)	12 (12)
	Some university credit, no degree	13 (29)	7 (7)
	Trade/technical/vocational training	2 (4)	11 (11)
	Bachelor’s degree	7 (16)	37 (38)
	Postgraduate degree	3 (7)	25 (26)
Employment	Unemployed/unable to work	3 (7)	2 (2)
	Full time employment	17 (38)	86 (89)
	Part time/casual employment	13 (29)	4 (4)
	Student	2 (4)	3 (3)
	Homemaker	7 (16)	1 (1)
	Retired	3 (7)	1 (1)
Ethnicity	Aboriginal/Torres Strait Islander	2 (4)	4 (4)
	Arab	0 (0)	0 (0)
	Asian	2 (4)	8 (8)
	Black/African	0 (0)	7 (7)
	Caucasian/Australian	32 (71)	53 (55)
	European	9 (20)	11 (11)
	Other	0 (0)	13 (13)
	Prefer not to say	0 (0)	1 (1)
Relationship status	Single/never married	27 (60)	9 (9)
	Married/domestic partnership	14 (31)	82 (85)
	Divorced/Separated	4 (9)	6 (6)
No. of children (under 18 years)	0	21 (47)	0 (0)
	1	15 (33)	11 (11)
	2	8 (18)	34 (35)
	3+	1 (2)	52 (54)
Previous engagement in a parenting program	Have previously engaged in a program	3 (7)	25 (26)
	Have not previously engaged in a program	42 (93)	72 (74)

**Table 2:** Parenting program attributes and attribute levels that appear in Experiment 1 and Experiment 2, respectively.

Experiment 1: Program Attribute	Experiment 2: Attribute Levels	Description
Program content	(a) General parenting information (b) Specific information about a child concern (c) Practical, hands-on activities	How general or specific, and hands-on the content is. Receiving general information about parenting, specific information about a child’s problem, engaging in hands-on learning activities, or receiving a mix of information and activities
Cost of the program	(a) $0 (b) $50 (c) $100	The cost of participating in the parenting program
Style of participation	(a) Individually (b) With other fathers (c) With other parents and carers	Choosing to participate in the program individually, in a group of other dads, or with your partner/co-parent
Delivery modality	(a) Face-to-face (b) Online (asynchronous) (c) Telehealth (synchronous)	Choosing to receive the program face-to-face, through a website, through an app, via video call (e.g., Zoom), or via SMS updates
* Facilitator qualities	N/A	The background experience and gender of the person facilitating the program
* Length of the program	N/A	The amount of time and sessions the program runs over

* attributes excluded in Experiment 2.

**Table 3:** Fathers’ preferences for parenting program attributes, as measured by average standardised scores.

	Program Content	Cost	Style of Participation	Delivery Modality	Facilitator Qualities	Program Length
Average standardised score	0.343	0.155	0.062	−0.006	−0.081	−0.419
Standard deviation	0.534	0.454	0.476	0.547	0.492	0.612

**Table 4:** Parameter estimates for the multinomial logistic regression exploring program content, cost, participation style, and delivery modality and fathers’ decision to engage in parenting programs.

Attribute	Level	Estimate	Robust *t*-Ratio	Robust *p*-Value (2-Sided)	Robust Standard Error
Cost	$0	0	-	-	-
	$50	−0.743	−7.597	<0.001	0.119
	$100	−1.699	−9.314	<0.001	0.288
Participation	Individually	0	-	-	-
	With dads	−0.126	−1.011	0.312	0.178
	With parents	−0.159	−1.360	0.173	0.149
Content	General information	0	-	-	-
	Specific information	0.357	3.310	<0.001	0.127
	Activities	0.501	3.449	<0.001	0.193
Delivery	Face-to-face	0	-	-	-
	Asynchronous	−0.701	−3.728	<0.001	0.256
	Synchronous	−0.786	−4.344	<0.001	0.267

Note. The first listed level of each attribute acted as the reference level, with utility set to 0.

**Table 5:** Top 10 Preferred Parenting Programs Based on Model-Predicted Choice Shares.

Cost	Delivery Modality	Program Content	Participation Style	*p*	Cumulative_*p*
$0	Face-to-face	Activities	Individually	0.034	0.034
$0	Face-to-face	Activities	With dads	0.033	0.067
$0	Face-to-face	Activities	With parents	0.032	0.099
$0	Face-to-face	Specific information	Individually	0.028	0.127
$0	Face-to-face	Specific information	With dads	0.028	0.155
$0	Face-to-face	Specific information	With parents	0.026	0.181
$0	Online	Activities	Individually	0.024	0.205
$0	Online	Activities	With dads	0.023	0.229
$0	Online	Activities	With parents	0.023	0.252
$0	Telehealth	Activities	Individually	0.022	0.273

Note. The order of the columns reflects that fathers have the strongest and most consistent preferences for attributes/levels towards the left margin, and weaker more variable preferences for attributes/levels toward the right margin.

**Table 6:** Top 10 Preferred Parenting Programs Based on Refined Model-Predicted Choice Shares Excluding Participation Style.

Cost	Delivery Modality	Program Content	*p*	Cumulative_*p*
$0	Face-to-face	Activities	0.099	0.098
$0	Face-to-face	Specific information	0.082	0.181
$0	Online	Activities	0.071	0.252
$0	Telehealth	Activities	0.063	0.315
$0	Online	Specific information	0.059	0.373
$0	Face-to-face	General information	0.056	0.429
$0	Telehealth	Specific information	0.052	0.481
$50	Face-to-face	Activities	0.049	0.531
$50	Face-to-face	Specific information	0.041	0.572
$0	Online	General information	0.040	0.612

Note. The order of the columns reflects that fathers have the strongest and most consistent preferences for attributes/levels towards the left margin, and weaker more variable preferences for attributes/levels toward the right margin.

## Data Availability

The data presented in this study are available on request from the corresponding author due to restrictions imposed by the Human Research Ethics Committee’s approval for this study.
